# Decision‐Support Framework for Nutritional Risk in Small Cell Lung Cancer: A Time‐Series Model Using Imputation Strategies for Incomplete Clinical Data

**DOI:** 10.1111/1759-7714.70381

**Published:** 2026-08-25

**Authors:** Ruili Pan, Yifan Zhao, Mengzhao Wang, Weida Liu, Shuo Ji, Ya Liu, Yuchen Zhang, Chenxi Ma, Minjiang Chen, Yan Xu

**Affiliations:** ^1^ Department of Nursing Peking Union Medical College Hospital, Chinese Academy of Medical Sciences and Peking Union Medical College Beijing China; ^2^ Clinical Research Validation Platform, National Facility for Translational Medicine, Peking Union Medical College Hospital, Chinese Academy of Medical Sciences and Peking Union Medical College Beijing China; ^3^ Department of Pulmonary and Critical Care Medicine Peking Union Medical College Hospital, Chinese Academy of Medical Sciences and Peking Union Medical College Beijing China; ^4^ Peking Union Medical College Hospital, State Key Laboratory for Complex Severe and Rare Diseases, Institute of Clinical Medicine, Chinese Academy of Medical Sciences Beijing China

**Keywords:** machine learning models, missing data imputation, predictive modeling, prognostic nutritional index (PNI), small cell lung cancer (SCLC)

## Abstract

In patients with small cell lung cancer (SCLC), nutritional status is a key determinant of disease progression, treatment tolerance, and prognosis. The prognostic nutritional index (PNI), reflecting both immune and nutritional conditions, is widely used to evaluate prognostic risk, but its longitudinal monitoring is often limited by incomplete clinical data in real‐world settings. This study aimed to propose a decision‐support framework for predicting future nutritional status and improving risk screening in SCLC patients with missing data. Using PNI values and related variables from the first four follow‐up time points to predict the PNI at the fifth time point (PNI5) and evaluated the impact of different missing data imputation strategies on predictive performance. Missing PNI values were imputed using mean imputation, multiple imputation (MI), Kalman filtering, k‐nearest neighbors (KNN), and XGBoost imputation. Predictive models were developed with two machine learning algorithms: random forest (RF) and XGBoost. The RF model demonstrated better overall performance, and MI combined with RF achieved the best predictive accuracy (MAE: 2.952; RMSE: 3.727). Predicted PNI values were further translated into risk categories using a predefined threshold (PNI = 45), with overall accuracy of 93.33%, PPV 95.24%, and NPV 92.59%. Importantly, the model enabled risk assessment in patients with incomplete laboratory data, covering previously unassessable cases and reducing monitoring gaps. This study highlights the importance of appropriate imputation strategies in and provides a practical tool for continuous nutritional risk assessment and early identification of high‐risk patients in SCLC.

## Graphical abstract

This study proposes a machine learning‐based decision‐support framework for SCLC nutritional risk prediction using incomplete longitudinal PNI data. MI combined with random forest outperformed four imputation strategies (MAE 2.952, RMSE 3.727). Predicted values were translated into risk categories (threshold = 45) with 93.33% accuracy, enabling early identification of high‐risk patients and early intervention.

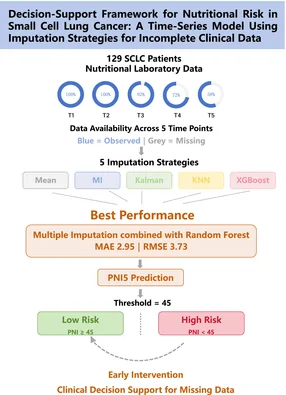

## 1 Introduction

The prognostic nutritional index (PNI) is an important indicator that comprehensively reflects the nutritional and immune status of patients. In clinical practice, PNI provides objective information for nutritional assessment and consistent patient monitoring [1, 2, 3, 4]. Small cell lung cancer (SCLC) is an aggressive subtype of lung cancer characterized by rapid progression and early dissemination. Malnutrition and immune impairment are common in these patients, often limiting treatment response and leading to poor nursing and clinical outcomes [5]. In recent years, the clinical value of PNI in SCLC has received increasing attention [6, 7]. Given the aggressive nature of SCLC, nutritional status is highly dynamic during treatment, and timely assessment is essential for guiding clinical management and treatment decision‐making.

Previous studies have shown that the dynamic trend of PNI provides a more accurate reflection of disease progression and treatment response than a single time‐point measurement. In particular, in the long‐term management of malignant tumors, dynamic changes in PNI (from preoperative to postoperative) are more effective in predicting patient prognosis compared with a one‐time preoperative measurement [8, 9]. However, despite the recognized importance of dynamic monitoring, the ability to anticipate future nutritional status based on longitudinal data remains limited, restricting proactive risk identification in clinical practice.

In real‐world clinical practice, this predictive modeling often faces multiple challenges, including interruptions in follow‐up, inconsistent testing intervals, and incomplete data collection, leading to the frequent presence of missing values. Missing data not only compromise the completeness and representativeness of a dataset but also distort the captured trajectory of a patient's nutritional evolution, thereby influencing risk estimation and the quality of clinical decision‐making [10, 11, 12]. Therefore, appropriate handling of missing data is a critical step in ensuring the quality of clinical prediction research.

In clinical research, different approaches to handling missing values may lead to entirely different predictive outcomes, particularly, in situations with limited sample sizes [13, 14] and unclear missing mechanisms [15, 16]. However, most existing studies primarily focus on statistical performance, with limited attention to how imputation strategies may influence downstream clinical decision‐making, particularly, in longitudinal monitoring scenarios.

Aiming to bridge the gap between data incompleteness and precise clinical forecasting, this study compared the predictive performance of various missing value imputation methods, including mean imputation, multiple imputation (MI), Kalman filtering, k‐nearest neighbors (KNN), and XGBoost. Based on the data from the first four follow‐up time points to predict PNI at the fifth time point (PNI5). This study provides practical decision‐support tool for nutritional risk assessment in clinical follow‐up and supports the continuity of evaluation in real‐world data settings.

## 2 Methods

This section describes the study design, data characteristics, missing data handling strategies, and modeling procedures. Specifically, the study design and data characteristics are first presented, followed by the calculation of PNI. The mechanisms of missing data are then outlined, and five imputation strategies are applied to handle missing values. Predictive models are then developed to estimate PNI at the final follow‐up, and their performance is evaluated. Finally, a clinically oriented decision‐support analysis is conducted by applying a predefined PNI threshold to translate predicted values into risk categories, enabling assessment of the model's utility in supporting nutritional risk identification.

### 2.1 Study Design and Data Characteristics

This retrospective study included 129 patients with advanced SCLC who received systemic treatment at the Lung Cancer Diagnosis and Treatment Center of the Department of Respiratory Medicine, Peking Union Medical College Hospital, Chinese Academy of Medical Sciences, between January 2019 and April 2023. The observation period was 12 months, and data were collected at five time points: before initiation of antitumor therapy (baseline), and at 3, 6, 9, and 12 months after treatment. All data were obtained from the hospital information system (HIS), including inpatient and outpatient follow‐up records, and covered patients' demographic characteristics, clinical features, and laboratory test results.

Inclusion criteria:

- Patients diagnosed with SCLC who received diagnosis and treatment at this hospital during the study period:
- Patients with partial or complete records required for PNI calculation and available variables for subsequent analysis.

Exclusion criteria:

- Patients with unclear diagnosis or concomitant other malignancies:
- Patients whose key variables (e.g., indicators required for PNI calculation) were missing at all time points, making analysis infeasible;
- Duplicate records or data with abnormal quality (e.g., obvious logical errors).

To minimize acute stress‐induced fluctuations in laboratory parameters (serum albumin and total lymphocyte count), all baseline and follow‐up blood samples for PNI assessment were collected during routine clinical visits when patients were afebrile and in a stable, nonacute stress condition.

**FIGURE 1:**
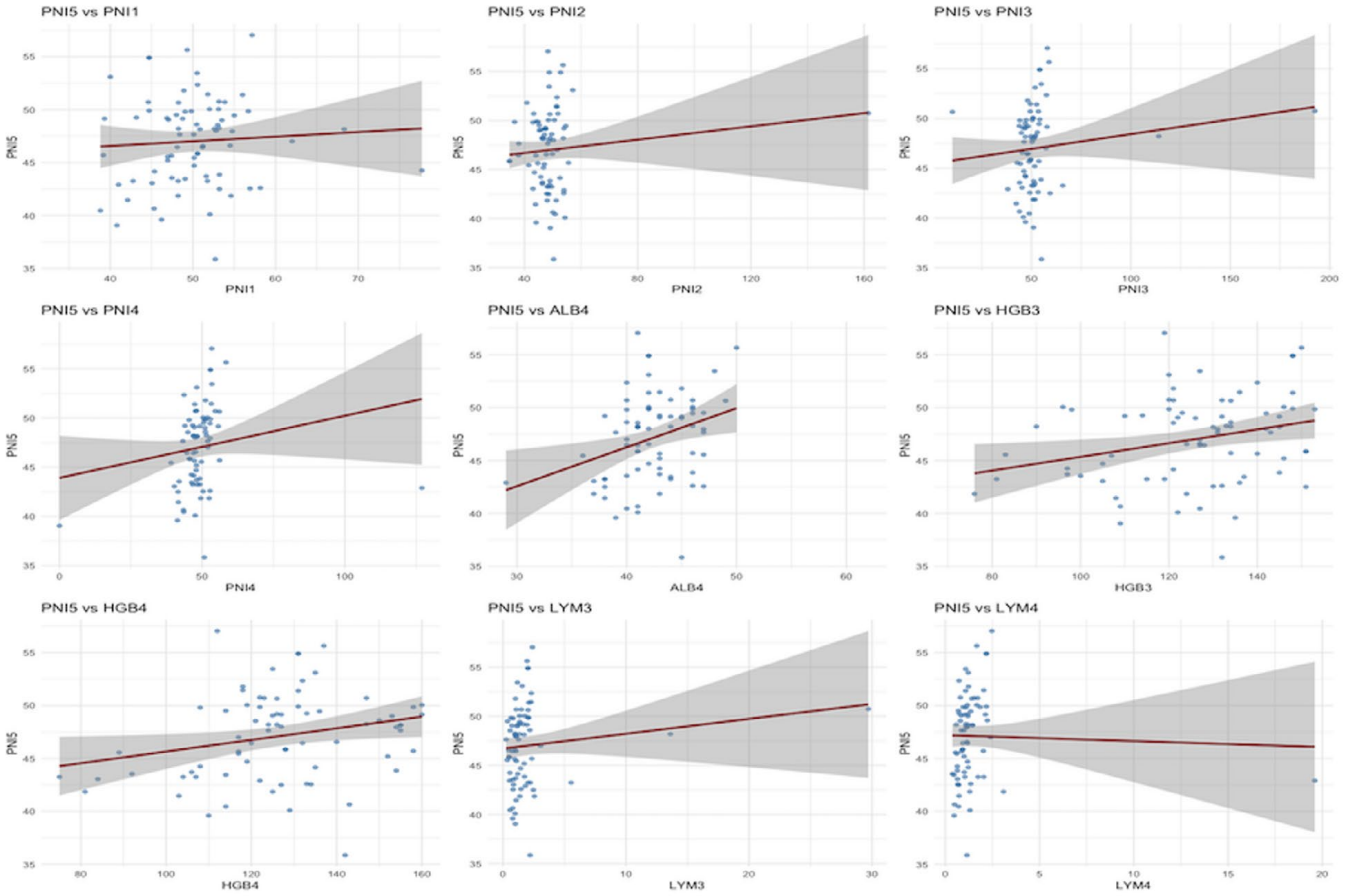
Scatter plot matrix of key variables.

During the preliminary exploratory analysis of candidate feature variables, scatter plots of correlations between variables were generated (Figure 1). The results indicated that the relationships between these variables and PNI were largely nonlinear with potential complex interactions, rather than simple linear associations. Based on this observation, nonlinear modeling approaches were considered for subsequent analysis.

To provide an overall understanding of the basic characteristics of the study data, this section presents only the fundamental information of the primary variables involved in subsequent analyses (Table 1). Detailed criteria for variable selection are described in subsequent sections. Furthermore, multivariable linear regression analysis was performed on the baseline dataset to assess potential baseline confounding effects (age and clinical stage, Stages III vs. IV) on baseline PNI values.

**TABLE 1:** Baseline characteristics and missing data proportions.

Variable	Median (IQR) or *n* (%)	Missing (%)
Demographic variables		
Sex, male	105 (81.3%)	0
Sex, female	24 (18.6%)	0
Age, years	61 (56–66)	0
Clinical stages		
Stage III	56 (43.4%)	0
Stage IV	73 (56.5%)	0
Lymphocyte count (×10^9^/L)		
LYM1	1.66 (1.23–2.13)	0
LYM2	1.21 (0.85–1.65)	0
LYM3	1.14 (0.79–1.74)	7
LYM4	1.11 (0.85–1.46)	28
LYM5	1.1 (0.70–1.46)	41
Albumin (g/L)		
ALB1	42 (39–44)	0
ALB2	41 (39–43)	0
ALB3	42 (40–44)	8
ALB4	42 (40–45)	28
ALB5	42 (39.5–43)	42
Prognostic nutritional index		
PNI1	50.15 (46.95–53.2)	0
PNI2	47.9 (45.1–50.21)	0
PNI3	48.85 (44.93–51.64)	8
PNI4	48.05 (45–51.48)	28
PNI5	47.95 (43.64–49.84)	42
Hemoglobin (g/L)		
HGB3	123 (108–134)	7
HGB4	127 (117–137.25)	28

*Note:* Numbers indicate time points: 1 = baseline, 2 = 3 months, 3 = 6 months, 4 = 9 months, 5 = 12 months.

Abbreviations: ALB = albumin; HGB = hemoglobin; LYM = lymphocyte count; PNI = prognostic nutritional index.

### 2.2 Calculation of PNI

In this study, the PNI was calculated using the following formula:

$$\begin{array}{c} \mathrm{PNI}=\text{serum albumin concentration}\;\left(g/L\right)\\ \;+5\times \text{peripheral blood lymphocyte count}\;\left(\times 10^{9}/L\right) \end{array}$$

This formula represents the SI unit conversion of the classic model proposed by Onodera et al. [17]. Although most early studies used PNI = 10 × serum albumin concentration (g/dL) + 0.005 × peripheral blood lymphocyte count (/mm^3^), the SI unit version was adopted in this study to align with the units of the collected data. This form has been widely applied in recent tumor and nutrition‐related studies and demonstrates good applicability and comparability [18, 19].

In this study, PNI values were calculated for each patient at five time points (PNI1–PNI5) for subsequent missing value imputation and data analysis.

### 2.3 Mechanisms of Missing Data

In multivariable longitudinal clinical data, missing values often occur across both time and variable dimensions, which may affect data analysis and model performance. According to Rubin's framework, missing data can be classified into three categories [20]:

- Missing completely at random (MCAR): missingness is unrelated to the variable itself or to any other observed or unobserved variables. For example, laboratory data (e.g., serum albumin) may be missing due to equipment failure.
- *Missing at random (MAR)*: missingness is related to other observed variables but not to the missing value itself. For example, older patients may be more likely to have missing height or weight measurements due to limited mobility.
- *Missing not at random (MNAR)*: missingness is related to the unobserved value itself or to unobserved factors. For example, patients with very poor nutritional status may refuse blood draws, resulting in missing nutritional indicators

In real‐world clinical follow‐up data, the mechanisms of missing values are often difficult to categorize clearly and are typically influenced by multiple factors, including patient compliance, timing of assessments, and data recording processes [21]. In this study, missing values in PNI were observed to cluster at certain time points and among specific patient groups, suggesting that the missingness is more likely to follow a MAR or even a MNAR mechanism. However, due to the lack of detailed contextual information on data collection, the exact mechanism could not be determined.

When the missing mechanism cannot be reliably determined, blindly applying methods based on strong assumptions may introduce systematic bias, thereby affecting the stability and interpretability of the model results. To evaluate different imputation approaches under real‐world data conditions, this study included commonly used methods such as mean imputation and KNN as reference approaches, as well as MI, XGBoost regression, and Kalman filtering. These methods were selected to enable comparison across different approaches in the presence of longitudinal data and incomplete observations.

#### 2.3.1 Strategy Overview for PNI Imputation

In this study, the PNI was measured at five time points (PNI1–PNI5), with missing values present in PNI1–PNI5. To evaluate the performance of different imputation methods, missing values in PNI1–PNI4 were first imputed using different approaches (see Section 2.4). The imputed PNI1–PNI4 values were then used as predictor variables, while PNI5 was treated as the outcome variable. The predictive values were then compared with the observed PNI5 to assess the relative performance of each imputation method in the actual dataset.

### 2.4 Imputation Strategies for Missing Data

#### 2.4.1 Statistical Methods

##### 2.4.1.1 Mean Imputation

Mean imputation replaces missing values with the mean of the observed values [22]. It assumes data are MCAR and does not account for relationships between variables [23, 24, 25]. In this study, mean imputation was used as a reference method for comparison.

##### 2.4.1.2 MI

MI with predictive mean matching (PMM) is commonly used when data are assumed to be MAR [26, 27, 28, 29, 30]. In this study, MI was applied to handle incomplete data and generate complete datasets for analysis. PMM was used within the MI framework to ensure that imputed values remained consistent with observed data. Five imputed datasets were generated using the mice package in R with a fixed random seed. The first imputed dataset was used for subsequent modeling and statistical analyses.

##### 2.4.1.3 Kalman Filter‐Based Imputation

Kalman filtering is a method for handling missing values in time series data [31]. It updates the estimates of missing values over time and keeps the data sequence continuous. In this study, we applied the StructTS‐based Kalman method for each variable to preserve its temporal characteristics.

#### 2.4.2 Machine Learning Methods

Machine learning methods are increasingly used in medical data analysis and can be applied to handle missing values. Compared with traditional approaches, these methods use information from observed data to estimate missing values and can account for nonlinear relationships between variables [32, 33, 34]. In this study, two commonly used methods, KNN and extreme gradient boosting (XGBoost), were selected for comparison.

##### 2.4.2.1 KNN Imputation

KNN imputation estimates missing values using information from similar observations [35, 36, 37]. In this study, all variables were standardized (mean = 0, SD = 1) before imputation to reduce the influence of different measurement scales. The imputation was performed with *k* = 5, and the results were then transformed back to the original scale.

##### 2.4.2.2 XGBoost Imputation

XGBoost is a machine learning algorithm that can model complex relationships between variables [38]. In this study, XGBoost regression was used to impute missing values in PNI1–4 and related variables. For each variable with missing data, a model was trained using the remaining complete variables as predictors, and missing values were estimated iteratively. The model was set with 50 iterations and the squared error as the objective function to ensure stable and accurate imputation.

### 2.5 Model Development and Evaluation

Although missing value imputation is a critical step in data preprocessing, it is often not the ultimate goal in most studies [33]. In clinical research, imputed data are typically used for subsequent analyses, such as prediction or outcome assessment. Therefore, the evaluation of imputation methods should consider their impact on downstream analyses.

In this study, the primary analytical task was to predict PNI at the final follow‐up (PNI5). Given its clinical relevance, relatively high missing rate, and the complexity of missingness, PNI5 was not directly imputed. Instead, PNI values from the first four time points (PNI1–PNI4) and related variables were imputed and standardized as needed and used as model inputs. Predictive models were then developed to estimate PNI5, and the predicted values were compared with the observed values. This approach was used to evaluate the performance of different imputation methods in a prediction setting.

#### 2.5.1 Modeling Approaches

Random forest (RF) and XGBoost (Extreme Gradient Boosting, XGB) are robust tree‐based ensemble algorithms widely used in clinical prediction modeling due to their superior ability to handle complex, nonlinear clinical data [38, 39, 40]. Given the limited sample size and the intricate relationships between variables in this study, both methods were employed to ensure the stability of the predictive framework. This approach allowed for a comprehensive evaluation of how different imputation strategies influence downstream nutritional risk forecasting in a real‐world oncology setting.

#### 2.5.2 Predictor and Outcome Variables

For model construction, PNI assessments from the first to fourth time points (PNI1–PNI4) were combined with several key physiological indicators: serum albumin at the fourth measurement (ALB4), hemoglobin at the third and fourth measurements (HGB3, HGB4), and lymphocyte counts at the third and fourth measurements (LYM3, LYM4). Together, these nine variables were used to predict PNI5.

Variable selection was based on both statistical results and clinical considerations. Variables with statistical significance (*p* < 0.05) were included, and additional variables related to nutritional and immune status were retained based on clinical relevance. To avoid information leakage, all variables from the fifth time point were excluded from model training.

During model development, the selected variables were used as input features, and PNI5 was used as the target variable for regression analysis. This design allowed evaluation of the impact of imputation on prediction performance.

#### 2.5.3 Model Performance Metrics

To evaluate model performance in predicting PNI5, mean absolute error (MAE) and root mean square error (RMSE) were used [33, 41]. MAE reflects the average magnitude of prediction error, while RMSE gives greater weight to larger errors. Using both metrics provides an assessment of prediction performance under different imputation strategies.

### 2.6 Clinical Decision‐Making Support Framework

Following the validation of predictive performance (MAE, RMSE), this study developed a clinical decision‐support framework to evaluate the model's utility and practical value in real‐world clinical practice scenarios. The transition from continuous numerical prediction to categorical discriminant analysis is essential, as clinical management is typically triggered by specific thresholds rather than raw scores. Validating the stability of the model near clinical cutoff points is critical for ensuring patient safety. The evaluation scheme is detailed as follows:

#### 2.6.1 Definition of the Clinical Decision Threshold

Based on clinical consensus and prior evidence [17, 42], a PNI value of 45 was established as the clinical cutoff for nutritional risk stratification. Accordingly, the predictive results were transformed into a binary classification: a PNI < 45 was defined as “High Risk,” serving as an early warning signal to initiate intensive nutritional support, while a PNI ≥ 45 was categorized as “Low Risk,” indicating a relatively stable nutritional status.

#### 2.6.2 Clinical Interpretation of Performance Metrics

To assess the effectiveness of the model in supporting clinical decisions, a confusion matrix was employed to evaluate classification performance near the clinical decision threshold [43]. Based on the confusion matrix, key performance metrics—including overall accuracy, positive predictive value (PPV), negative predictive value (NPV), and sensitivity—were calculated to quantify the model's ability to identify nutritional risk. The metrics are defined as follows:

- *Overall accuracy*: This metric evaluates the model's comprehensive ability to make correct judgments. Clinically, it indicates how consistently the automated screening aligns with a nurse's manual, precise assessment. High accuracy ensures that the tool can effectively streamline the assessment process, reducing the cognitive burden of routine calculations and allowing nurses to prioritize direct patient care.
- *PPV*: This evaluates the precision and clinical credibility of the early warning system. A high PPV indicates that nutritional risk alerts issued by the model are highly reliable, significantly reducing “alarm fatigue” caused by frequent false positives (FPs) (i.e., instances where a risk is flagged for a clinically stable patient). This ensures that clinical resources and interventions are precisely targeted toward the population genuinely at risk.
- *NPV*: This evaluates the safety margin of the screening process. A high NPV gives clinicians confidence that a “Low Risk” result is genuinely safe and reliable. If the NPV is low, it means some high‐risk patients might be incorrectly missed or “flagged as normal.” This could lead to a delay in starting necessary nutritional support, allowing potential complications to go unnoticed. Maintaining a strong NPV is essential to prevent these intervention delays and ensure that the patient's monitoring chain stays unbroken and safe.
- *Sensitivity*: This measures the model's completeness in capturing patients with potential nutritional risks. High sensitivity means a lower “miss rate” for at‐risk populations. It ensures that patients on the brink of malnutrition are identified by the system in a timely manner, preventing evaluation lags and the omission of optimal clinical intervention windows.

Additionally, consistency scatter plots were used to visualize the agreement between predicted and observed PNI values, as well as the distribution of samples around the clinical decision threshold.

#### 2.6.3 Assessment of Continuity Recovery in Nutritional Monitoring

To address the “monitoring gaps” in clinical practice—where clinicians are unable to manually calculate the PNI due to incomplete laboratory data—this study introduced Assessment Coverage to measure the model's clinical recovery capability. This metric focuses on patients who were previously “unassessable” due to missing values. By applying the model to determine risk status based on available clinical features, the proportion of patients regaining a definitive risk classification was calculated. This transition from “unassessable” to “automated alert” essentially leverages algorithmic capabilities to fill monitoring blind spots, ensuring that the nutritional assessment chain remains uninterrupted throughout the patient's follow‐up [44].

## 3 Results

Prior to evaluating model predictions, a multivariable linear regression analysis was performed to assess potential confounding effects of age and clinical stage (Stage IV vs. Stage III) on baseline PNI. The overall model was not statistically significant (*F* = 0.3949, *p* = 0.6746). Neither age (*β* = 0.0024, *t* = 0.040, *p* = 0.968) nor clinical stage (*β* = −1.0166, *t* = −0.889, *p* = 0.376) showed a significant association with baseline PNI, indicating that baseline PNI was independent of these demographic and clinical parameters in this cohort.

### 3.1 Evaluation of Model Predictive Accuracy and Error Distribution

#### 3.1.1 Numerical Error Analysis Across Imputation Strategies

**FIGURE 2:**
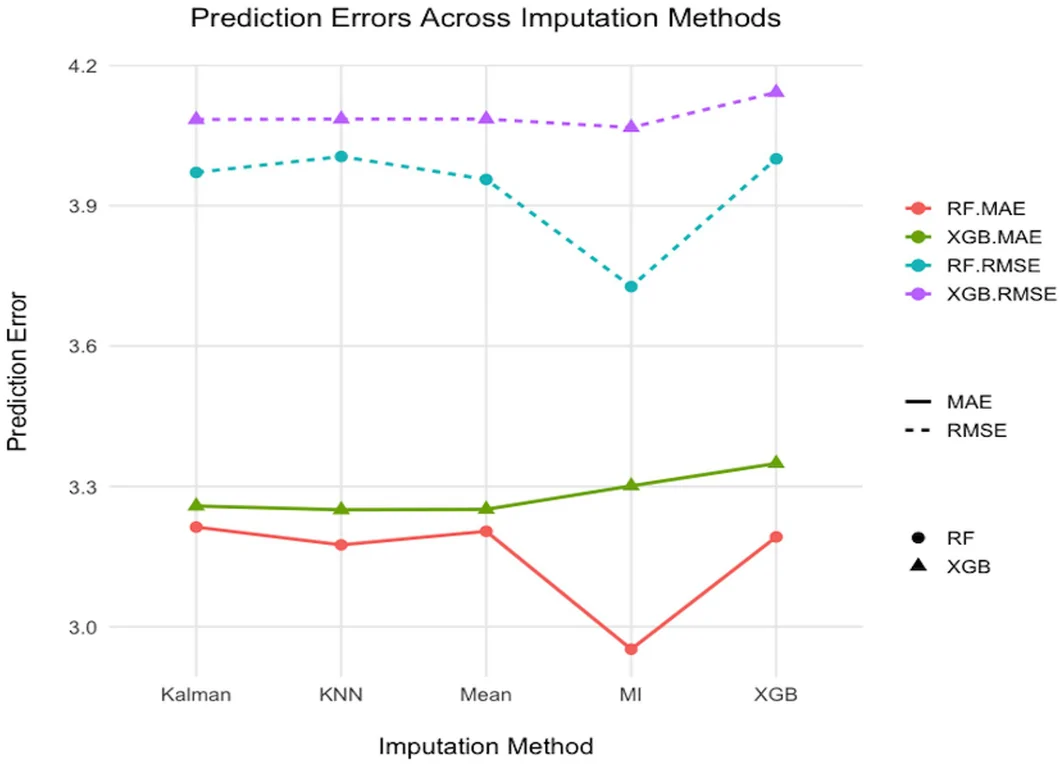
Prediction performance under different imputation strategies in random forest and XGBoost models.

First, the MAE and RMSE values of each imputation method under the two modeling strategies are summarized (Table 2), along with line plots (Figure 2), to compare the predictive performance of different imputation approaches for PNI5.

**TABLE 2:** Prediction performance under different imputation strategies.

Model	Method	MAE	RMSE
Random forest	Mean	3.204	3.956
	MI	2.952	3.727
	Kalman	3.213	3.971
	KNN	3.175	4.005
	XGB	3.192	4.000
XGBoost	Mean	3.251	4.085
	MI	3.301	4.067
	Kalman	3.258	4.084
	KNN	3.250	4.085
	XGB	3.349	4.142

Overall, the various imputation methods showed similar performance in terms of MAE and RMSE, with only minor differences. Across all methods, both MAE and RMSE were consistently lower under the RF model than under XGBoost, suggesting better predictive performance in this dataset. Within the RF model, MI achieved the lowest MAE (2.952) and RMSE (3.727), indicating the smallest prediction error among the evaluated methods. KNN imputation showed a similar MAE (3.175) but a slightly higher RMSE (4.005). XGB, mean, and Kalman imputations yielded slightly higher errors (MAE = 3.192–3.213; RMSE = 3.956–4.000).

In contrast, all imputation methods produced higher errors in the XGBoost model. Notably, the lowest error in the XGBoost framework (KNN, MAE = 3.250; RMSE = 4.085) still exceeded the highest MAE observed under RF. Moreover, MAE values across the imputation methods in XGBoost were close (3.250–3.349), with similarly narrow RMSE ranges (4.067–4.142), indicating limited variation across methods within this model. Overall, while the RF model demonstrated lower prediction errors, the MI strategy consistently achieved the best performance among the evaluated imputation approaches.

#### 3.1.2 Visual Analysis of Prediction Bias and Distribution

**FIGURE 3:**
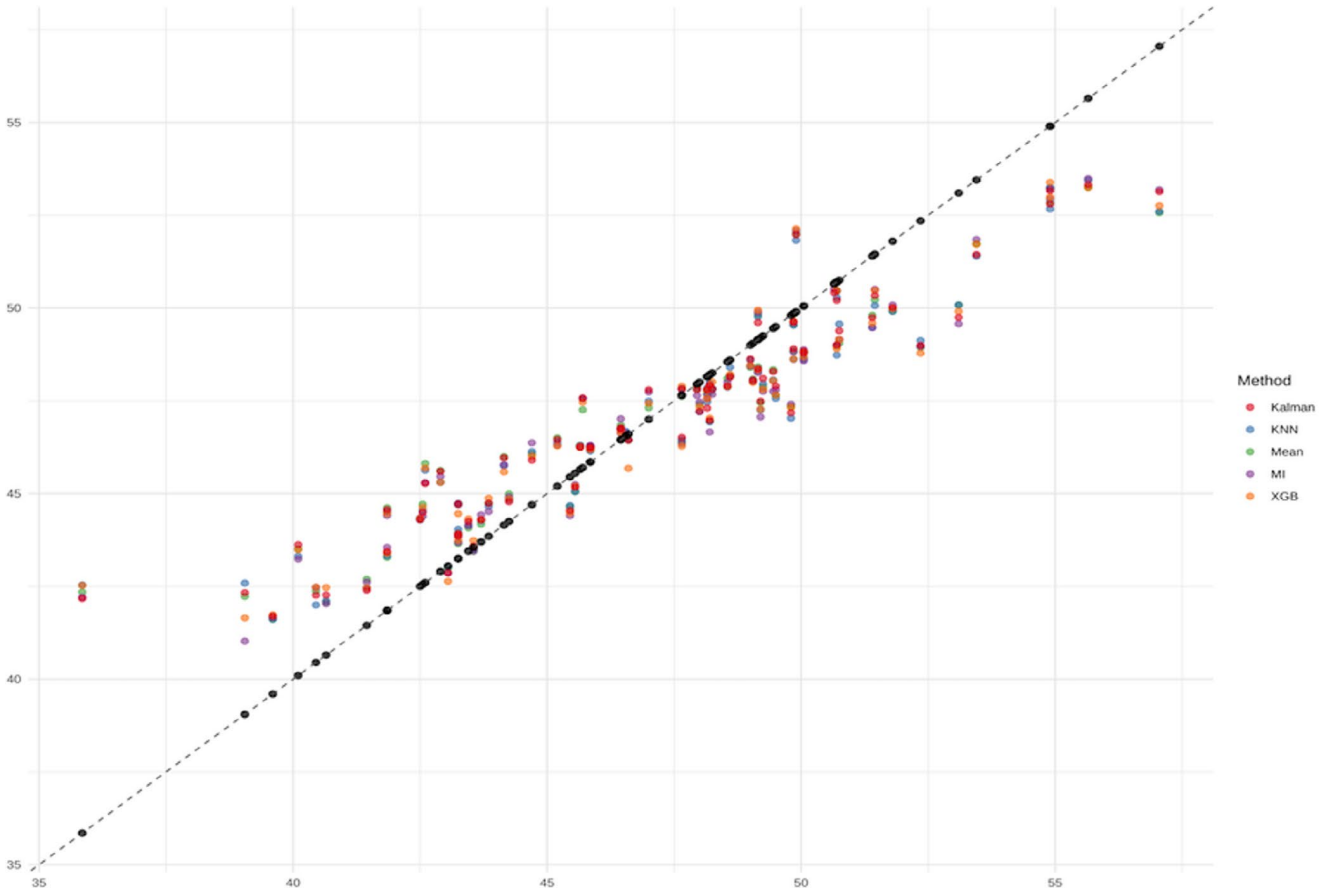
Scatter plot of predicted versus observed values for different imputation methods under the random forest model.

To further assess predictive performance, scatter plots of predicted versus observed PNI5 values under the RF model were generated for each imputation method (Figure 3). In the plots, each black point represents the observed value of a sample, while colored points indicate predicted values obtained using different imputation methods. A reference line of *y* = *x* is included to represent the ideal prediction scenario, where predicted values perfectly match the observed values. This reference line serves as an important benchmark for assessing the direction of deviation: points above the line indicate overestimation, whereas points below indicate underestimation.

As shown in the figure, the observed PNI5 values are primarily concentrated in the range of approximately 40–55, exhibiting a relatively clustered distribution that generally aligns along the *y* = *x* reference line. In contrast, the predicted values from different imputation methods show varying degrees of deviation from this line, indicating the presence of prediction error. Specifically, for observed values in the 45–50 range, the predictions are generally close to the true values, with points densely clustered around the reference line, suggesting that the model achieves a good fit and relatively small prediction errors within this interval. When the observed values are below 45, most points lie above the reference line, indicating that the predicted values are generally higher than the true values. This overestimation is particularly pronounced in the PNI5 range below 40, where predictions clearly exceed the observed values. When the observed values exceed 50, the predicted values are mostly below the reference line, reflecting a systematic underestimation in the higher value range. These patterns indicate that prediction accuracy varies across the range of PNI5 values, with better agreement in the mid‐range and greater deviation at the lower and higher ends.

### 3.2 Nutritional Risk Discrimination and Clinical Decision Support

Based on the confusion matrix analysis of the validation set (*N* = 75), the performance of the MI‐RF model in identifying nutritional risk among SCLC patients is presented.

#### 3.2.1 Evaluation of Decision Consistency

**FIGURE 4:**
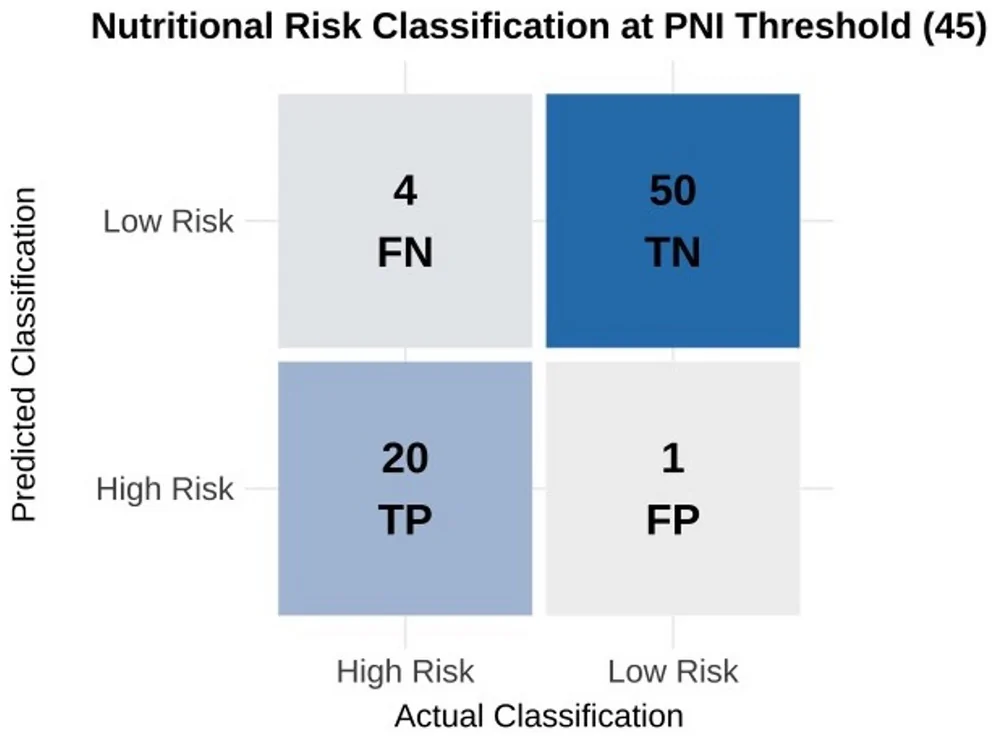
Nutritional risk classification at PNI threshold (45).

At the clinical intervention threshold of PNI = 45, the model achieved an overall accuracy of 93.33% (70/75). The classification performance is illustrated in the confusion matrix (Figure 4). Within the validation cohort, the model identified 20 true positive (TP) cases of nutritional risk and classified 50 cases as true negatives (TN), showing agreement with the reference classification based on complete laboratory data.

#### 3.2.2 Predictive Precision and Risk Identification

Among the 21 cases classified as high risk by the model, 20 were confirmed as TP and 1 as a FP, resulting in a PPV of 95.24%. The model identified 20 of 24 patients with actual nutritional risk, with four false negatives (FNs) cases, corresponding to a Sensitivity of 83.33%. Among the 54 cases classified as nonrisk, 50 were TNs, yielding a NPV of 92.59%.

#### 3.2.3 Recovery of Monitoring Vacuums

For the 54 samples where manual PNI scoring was interrupted due to missing laboratory indicators, the MI‐RF model achieved 100% coverage for risk qualitative assessment. In this specific subset, all model‐discriminated results indicated a low‐risk status, providing classification results for cases where manual assessment was not available.

## 4 Discussion

In patients with SCLC, maintaining continuous nutritional assessment during treatment is often challenging in routine practice. Although the PNI is widely used to reflect nutritional and immune status, its calculation depends on complete laboratory data, which are not always available due to missed visits or poor patient condition. This results in interruptions in longitudinal evaluation and may affect timely identification of nutritional risk. In this study, we addressed this clinically relevant limitation by integrating missing data imputation with longitudinal prediction of PNI, and further translating predicted values into clinically meaningful risk categories based on a predefined threshold, thereby enabling continuous nutritional risk assessment even in the presence of incomplete data.

In terms of prediction performance, the RF model generally outperformed XGBoost, and the combination of MI and RF achieved the lowest error (MAE: 2.952; RMSE: 3.727). This suggests that, for this type of longitudinal clinical data, appropriate handling of missing values is important for maintaining prediction stability. Previous studies have shown that MI provides less biased and more efficient estimates than traditional approaches such as complete‐case analysis [45, 46, 47], while RF models show strong robustness in handling complex and nonlinear clinical data structures [48]. Importantly, beyond numerical accuracy, the model demonstrated strong agreement with clinically defined risk classification at the PNI threshold (45), with high overall accuracy, PPV, and NPV, supporting its potential utility in clinical risk stratification.

The scatter plot analysis showed that prediction accuracy was not consistent across all value ranges. The model performed well in the mid‐range (approximately 45–50), where most observations were concentrated, but tended to overestimate lower values and underestimate higher ones. This pattern is consistent with the well‐recognized regression‐to‐the‐mean phenomenon in predictive modeling and reflects potential variability in model performance across different value ranges [49, 50]. In clinical settings, these extreme values often correspond to significant changes in nutritional status. Therefore, although the model performs well for general risk screening, predictions at extreme PNI values should be interpreted with caution, particularly when they may influence clinical decisions regarding the timing or intensity of nutritional intervention.

From a clinical perspective, the main value of this model lies in maintaining continuity of nutritional monitoring. Continuous nutritional assessment is recommended as a key component of cancer care [44, 51]. By enabling risk classification under such conditions, the model helps reduce gaps in longitudinal evaluation and allows clinicians to retain an overall understanding of a patient's nutritional trajectory. This may facilitate earlier identification of patients at potential nutritional risk and support clinical decision‐making, such as closer monitoring or consideration of nutritional support when indicated. However, consistent with current perspectives on artificial intelligence in healthcare, the model should be used as an aid to clinical judgment rather than a substitute (high‐performance medicine: the convergence of human and artificial intelligence).

### 4.1 Limitations

Several limitations should be noted. First, the sample size was relatively small, and extreme PNI values were limited, which may affect model performance at the boundaries. Second, this was a single‐center study, and external validation with larger, multicenter datasets is needed. Third, although key laboratory indicators were included, additional variables such as inflammatory markers, treatment response, and patient‐reported symptoms may further improve the model. Fourth, higher loss to follow‐up at 9 and 12 months—potentially confounded by unrecorded deaths or disease progression—may introduce potential bias into the longitudinal imputation. Future studies incorporating more comprehensive clinical‐related data may enhance its usefulness in real‐world monitoring and assessment.

## 5 Conclusion

In this study, we evaluated several imputation strategies for handling missing longitudinal data and assessed their impact on predicting PNI at the final follow‐up in patients with SCLC. The results showed that the combination of MI and RF achieved the best predictive performance (MAE: 2.952, RMSE: 3.727). When applied to the clinical threshold of PNI = 45, this model demonstrated strong consistency with observed risk classifications, supporting its use as a tool for identifying patients at nutritional risk.

The primary value of this approach lies in its ability to fill the gaps created by missing laboratory results. By providing estimated PNI values, the model helps clinicians maintain a continuous view of a patient's nutritional trend, rather than leaving those periods unassessed. While further validation is necessary, our findings suggest that integrating imputation with machine learning can act as a practical backup for nutritional monitoring in clinical practice, helping ensure that a patient's risk doesn't go unnoticed between scheduled visits.

## Author Contributions

**Ruili Pan:** investigation, supervision, methodology, writing – original draft, data curation. **Yuchen Zhang:** investigation, resources. **Chenxi Ma:** investigation, resources. **Ya Liu:** validation, resources. **Yan Xu:** conceptualization, project administration, supervision, writing – review and editing. **Yifan Zhao:** writing – original draft, writing – review and editing, formal analysis, methodology, data curation. **Shuo Ji:** validation, resources. **Weida Liu:** visualization, software, data curation. **Minjiang Chen:** supervision, project administration, conceptualization, writing – review and editing. **Mengzhao Wang:** funding acquisition, resources.

## Funding

This study was supported by CAMS Innovation Fund for Medical Sciences (CIFMS) (Grant 2024‐I2M‐C&T‐B‐006).

## Ethics Statement

This study's protocol was approved by the Ethics Committee of the hospital (I‐24YSB1890) and was conducted in accordance with the 1964 Helsinki Declaration and its later amendments or comparable ethical standards.

## Consent

All participants provided written informed consent.

## Conflicts of Interest

The authors declare no conflicts of interest.

## Data Availability

The data that support the findings of this study are available on request from the corresponding author, M.C (minjiangchen@163.com) and X.Y (maraxu@163.com). The data are not publicly available due to their containing information that could compromise the privacy of research participants.
